# Supplementary material for: A Reassessment of the Therapeutic Potential of a Dopamine Receptor 2 Agonist (D2-AG) in Endometriosis by Comparison against a Standardized Antiangiogenic Treatment
Source: Biomedicines. 2021 Mar 8;9(3):269. doi: 10.3390/biomedicines9030269 (PMC8001569; doi:10.3390/biomedicines9030269)
Supplement: Supplementary file 1 [file biomedicines-09-00269-s001.pdf]

biomedicines

Supplementary Material

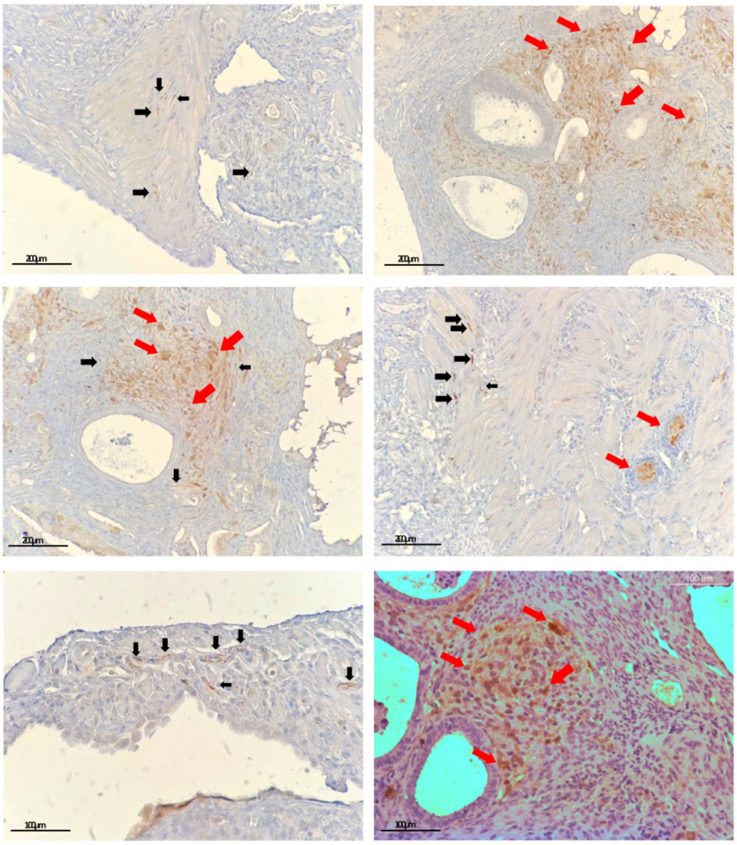

Figure S1. Pictures show representative images of xenografted human lesions stained against PGP 9.5, a putative specific marker of nerve fibers–note how PGP 9.5 ab recognizes structures compatible with the axon of nerve fibres (black arrows) but also whole cells (red arrows). Further confirmation of promiscuous expression PGP 9.5 is denoted by staining in complete areas of stromal and luminal compartment (red arrows).
